# Sex differences in patterns of white matter neuroplasticity after balance training in young adults

**DOI:** 10.3389/fnhum.2024.1432830

**Published:** 2024-08-27

**Authors:** Eric D. Kirby, Justin W. Andrushko, Lara A. Boyd, Karl Koschutnig, Ryan C. N. D’Arcy

**Affiliations:** ^1^BrainNet, Health and Technology District, Surrey, BC, Canada; ^2^Faculty of Individualized Interdisciplinary Studies, Simon Fraser University, Burnaby, BC, Canada; ^3^Faculty of Science, Simon Fraser University, Burnaby, BC, Canada; ^4^Djavad Mowafaghian Center for Brain Health, Faculty of Medicine, University of British Columbia, Vancouver, BC, Canada; ^5^Department of Sport, Exercise and Rehabilitation, Faculty of Health and Life Sciences, Northumbria University, Newcastle upon Tyne, Tyne and Wear, United Kingdom; ^6^Brain Behavior Laboratory, Department of Physical Therapy, Faculty of Medicine, University of British Columbia, Vancouver, BC, Canada; ^7^Institute of Psychology, BioTechMed Graz, University of Graz, Graz, Austria; ^8^Faculty of Applied Sciences, Simon Fraser University, Burnaby, BC, Canada

**Keywords:** white matter, neuroplasticity, motor learning, correlational tractography, MRI

## Abstract

**Introduction:**

In past work we demonstrated different patterns of white matter (WM) plasticity in females versus males associated with learning a lab-based unilateral motor skill. However, this work was completed in neurologically intact older adults. The current manuscript sought to replicate and expand upon these WM findings in two ways: (1) we investigated biological sex differences in neurologically intact young adults, and (2) participants learned a dynamic full-body balance task.

**Methods:**

24 participants (14 female, 10 male) participated in the balance training intervention, and 28 were matched controls (16 female, 12 male). Correlational tractography was used to analyze changes in WM from pre- to post-training.

**Results:**

Both females and males demonstrated skill acquisition, yet there were significant differences in measures of WM between females and males. These data support a growing body of evidence suggesting that females exhibit increased WM neuroplasticity changes relative to males despite comparable changes in motor behavior (e.g., balance).

**Discussion:**

The biological sex differences reported here may represent an important factor to consider in both basic research (e.g., collapsing across females and males) as well as future clinical studies of neuroplasticity associated with motor function (e.g., tailored rehabilitation approaches).

## 1 Introduction

The human brain can be categorized into two main tissue types: gray matter (GM: cell bodies (somas) that functions to receive and process information), and white matter (WM: axon projections that functions to transmit nerve signals between GM regions or between GM and the spinal cord). Neuroplasticity-related changes in these tissues can occur due to development, disease, degeneration, or be associated with learning and experiences. In essence, neuroplasticity can be operationally defined as a lifelong process where the brain reorganizes neural networks (Sampaio-Baptista and Johansen-Berg, 2017; Fields, 2010; Guglielman, 2012; Kesselring, 2015). Historically, activity-dependent changes in these tissues have mainly focused on changes at the synapse of neurons, located in GM. However, our group and others have demonstrated that neuroplasticity can occur beyond the synapse and along the axon in WM (Sampaio-Baptista and Johansen-Berg, 2017; Frizzell et al., 2021; Kirby et al., 2022; Reid et al., 2017; Weber et al., 2019).

Magnetic resonance imaging (MRI) derived techniques are commonly used to non-invasively examine and track structural and functional changes in the brain (Hamaide et al., 2016; Sale et al., 2017; Reid et al., 2017; Tardif et al., 2016). More specifically, MRI-derived techniques such as diffusion-weighted imaging (DWI) and myelin-water imaging (MWI) have been used extensively to focus MRI-based research on WM characterization, development, degeneration, and neuroplasticity (Kirby et al., 2022; Frizzell et al., 2021; Reid et al., 2017; Sale et al., 2017; Scholz et al., 2009; Islam et al., 2020; Weber et al., 2019; Lakhani et al., 2016).

Much of this previous literature acquires these *in-vivo* microstructural measures from each voxel of an MRI image. Averages of these values are usually taken across multiple voxels making up regions of interests [ROIs; (Lakhani et al., 2016)]. However, these ROI analyses may be skewed by non-population-specific atlases or by the inclusion of multiple fiber pathways within a voxel or ROI. Additionally, tract-based analyses that determine a whole tract mean measure may lead to less specificity and diminish sensitivity to detect structural properties and change. Previous literature has used tractography, computing individual’s WM tracts to then determine tract profiles (Kirby et al., 2022; Deng et al., 2018; Dayan et al., 2015; Yeatman et al., 2012; Baumeister et al., 2020). Tract profiles are helpful in determining the potential locus of change along a tract. New insights into WM change can be revealed using tract profiles that are not obvious from mean measures of that tract (Yeatman et al., 2012). These techniques have been implemented in neurologically intact individuals (Baumeister et al., 2020; Kirby et al., 2022), and in pathological populations, such as those effected by multiple sclerosis [MS; (Reich et al., 2007; Dayan et al., 2015)], Alzheimer’s disease (Zhang et al., 2019), and Parkinson’s disease (Cousineau et al., 2017). While this technique is a step in the right direction, it inherently relies on voxel-wise measurements and averages along fiber pathways, potentially diminishing sensitivity to detect change.

A tractography modality termed correlational tractography [grounded in connectometry (Yeh et al., 2016)] has been recently introduced to investigate WM tracts. This technique is based on a model-free tractography method that tracks connectivity with quantitative anisotropy (QA: the amount of anisotropic spins that diffuse along the fiber orientation) using the local connectome [the degree of connectivity between adjacent voxels in WM (Yeh et al., 2016)]. The core hypothesis in connectometry is that the associations between local connectomes and study variables tend to propagate along a common fiber pathway. Accordingly, this method focuses on tracking the difference instead of finding the difference in the tract (Yeh et al., 2016). Its primary measure, QA, is associated with axonal density (Yeh et al., 2010; Yeh et al., 2013b) and has majorly focused on neurological injury, showing decreases in QA associated with axonal loss or damage (Yeh et al., 2019b; Shen et al., 2015). Tractography based on QA is robust against peritumoral edema which contributes to more reliable results (Zhang et al., 2013) and is less sensitive to partial volume effects when compared to tractography techniques using FA, generalized FA (GFA), and anatomy in both phantom and *in-vivo* analyses (Yeh et al., 2013b). Subsequently, correlational tractography has been applied to cross-sectional analyses of WM tracts to correlate findings in neuropsychological disorders (Hula et al., 2020; Yeh et al., 2013a; Delaparte et al., 2017; Wang et al., 2019; Dresang et al., 2021; Brownsett et al., 2024) and neurodegenerative diseases (Sanchez-Catasus et al., 2021, 2022; Ghazi Sherbaf et al., 2018; Mojtahed Zadeh et al., 2018). This new method has also tracked longitudinal WM alterations at a group level, achieving higher sensitivity and specificity than conventional tractography (Yeh et al., 2016). Regarding longitudinal tracking, correlational tractography has been used primarily to index degeneration and neuronal damage from mild traumatic brain injury (Huang et al., 2023; Li et al., 2022). To our knowledge, this technique has not been used to study longitudinal changes facilitated by motor skill acquisition and learning.

Biological sex differences may influence the mechanisms that underly neuroplasticity (Marrocco and McEwen, 2016; McEwen and Milner, 2017). Differences in overall brain size, as well as in focused major brain structures was a popular area of research in early neuroimaging studies comparing females and males (Agartz et al., 1992; Coffey et al., 1998; Allen et al., 2002; Luders and Kurth, 2020). More specifically, microstructural values of WM tissue, such as DWI-derived fractional anisotropy (FA: a measure of anisotropy of water molecules’ diffusion in the brain), have been used to study microstructural differences in females and males (Shin et al., 2005; Westerhausen et al., 2003; Schmithorst et al., 2008; Liu et al., 2010; Björnholm et al., 2017; Hasan et al., 2008; Ritchie et al., 2018; Takao et al., 2014). These findings include female and male differences in developmental, degenerative, and experience-dependent changes in microstructural measures (Schmithorst et al., 2008; Toschi et al., 2020; Kirby et al., 2024).

Functionally, sex differences have also been noted in GM activation [i.e. the blood oxygen-level dependent (BOLD) signal] during voluntary motor tasks with males showing a larger surface area and higher variability in the BOLD signal during voluntary motor tasks (Andrushko et al., 2023). We have also shown differences between female and male-specific changes in WM following complex motor skill acquisition, despite comparable change in motor behavior (Kirby et al., 2024). Taken together, this work suggested biological sex is an important variable with demonstrable differences on both GM and WM during motor tasks and learning, which are not explained by differences in performance.

In our previous work, we studied a cohort of older adults [mean age (standard deviation): 64.2 (8.5) years] in a lab-based setting where they performed a unilateral reaching task (Kirby et al., 2024); as such it is unclear whether similar effects apply to different ages and/or motor learning tasks. While past work has shown relationships between DWI-derived measures in WM, with measures of motor learning (Frizzell et al., 2020, 2021; Kirby et al., 2022; Sale et al., 2017; Reid et al., 2017; Lakhani et al., 2016; Azzarito et al., 2021; Moore et al., 2017; Taubert et al., 2010; Borich et al., 2014) very little work has considered how biological sex impacts this relationship. Filling this knowledge gap will improve our understanding of how biological sex impacts neuroplastic change during motor learning and increase awareness of how WM disease might impact females versus males differently. A better understanding can improve monitoring of disease progressions that show differences between females and males (DeCasien et al., 2022; Ferretti et al., 2018). Moving forward with rehabilitation involves understanding the differences of pharmaceutical drug benefits and risks between females and males (Wierenga et al., 2024; Carey et al., 2017), as hormonal or sex-specific genotype interactions may affect drug metabolism and treatment response (Shulman, 2007; Abel et al., 2010; DeCasien et al., 2022; Ferretti et al., 2018).

Accordingly, the current study sought to extend work investigating differences in WM plasticity to consider younger adults learning a complex, whole body balance task. Given prior uses of correlational tractography and previous neuroimaging characterization of WM in females and males, our primary hypothesis was that females and males would show different patterns of WM change in widespread WM tracts, specifically reflected in greater increases in QA in females as compared to males.

## 2 Materials and methods

### 2.1 Experimental paradigm

The current study analyzed data from OpenNeuro [OpenNeuro Dataset ds003138, “*Tidying Up White Matter: Neuroplastic Transformations in Sensorimotor Tracts following Slackline Skill Acquisition*” (Koschutnig et al., 2023)]. A total of 60 participants were originally recruited for the study. After dropouts (7) and noisy data (1), a total of 52 participants [24 balance training (14 female) and 28 controls (16 female)] were included [mean age (standard deviation): 23.88 (3.48) years]. A fuller demographic breakdown is included in Table 1. Participants engaged in a slackline motor skill acquisition paradigm. Briefly, this began with an MRI scan before their first training session. Participants then worked with a professional slackline trainer for 90-minute sessions spaced approximately one week apart until they had reached a specified skill level. All participants reached the specified skill level in three or fewer sessions [range = 2–3 sessions; mean (standard deviation) = 2.29 (0.45)]. This included balancing on the slackline for one minute, followed by walking forward one meter and then backwards one meter. Once this skill level was reached, participants took part in a second MRI scan within 24 hours. The current study focused on pre- and post-training timepoints. All participants also underwent a follow-up MRI scan three weeks after training, but this was not included in the current analysis. All control participants were scanned following a similar timetable but did not undergo balance training.

**TABLE 1:** Demographic breakdown.

Group	Age [mean (standard deviation); range]	Pre-existing slackline experience [mean (standard deviation); range]
All participants	23.88 (3.48); 18 to 36 years	2.27 (1.16); 1 to 5
Balance trained female participants	22.50 (2.99); 19 to 28 years	2.07 (1.16); 1 to 5
Balance trained male participants	22.20 (2.14); 19 to 26 years	2.8 (1.25); 1 to 5
Control female participants	24.56 (3.16); 18 to 31 years	2.38 (1.11); 1 to 5
Control male participants	26.00 (3.89); 21 to 36 years	1.92 (0.95); 1 to 4

### 2.2 MRI acquisition

MRI data were acquired at the MRI-Lab Graz, Austria on a 3-Tesla (3T) Magnetom Skyra scanner (Siemens Healthineers, Erlangen, Germany) using a 32-channel head coil. All scans included a T1-weighted MPRage-Sequence (TR = 2400ms, TE = 2.26ms, 288 slices, thickness 0.8 mm, flip angle = 8°). Multi-shell DWI data were acquired with a multi-band accelerated echo-planar imaging sequence protocol (TR = 3500 ms, 68 slices, voxel size = 2 mm isotropic, multiband factor 4) with an anterior-posterior phase encoding direction for three b-values (b-values = 1000, 2000, and 3000 s/mm^2^; TE = 104ms, 113ms, 125ms; number of directions = 20, 30, and 64). Additionally, one b0-image and one extra b0-image in the reverse phase-encoding direction for each b-value was also acquired to correct for magnetic susceptibility-induced distortions. Overall, the total time of acquisition was about 14 minutes.

### 2.3 MRI processing

DWI data were corrected for echo planar imaging (EPI) distortions (Andersson et al., 2012), as well as motion and eddy corrected (Andersson and Sotiropoulos, 2016) using FSL’s *Top-up* and *Eddy* tools. Correlational tractography was prepared and carried out following DSI Studio (version: December 14, 2023). This technique is based on a model-free tractography method that calculates the spin distribution function (SDF: an orientation distribution function defined as the density of diffusing spins that have a displacement oriented at a direction during the diffusion time) (Yeh et al., 2010; Yeh and Tseng, 2011) on a local connectome scale (Yeh et al., 2016). By considering the possibility of more than one principal diffusion direction, model-free tractography methods are less susceptible to the partial volume of crossing fibers. The measurement at the base of connectometry, QA, is robust, specifically, to the free water effect (Yeh et al., 2013b). The difference between QA and regular orientation distribution function-based measures is that QA scales with spin density and the isotropic component is discarded (regular orientation distribution function is often min-max scaled to 0–1) (Yeh et al., 2013b). QA is a metric for each resolved fiber population, unlike FA or generalized FA (GFA), which is a metric for each voxel (Yeh et al., 2010). These attributes of QA, specifically the consideration of restricted diffusion, make it less affected by edema than FA and axial diffusivity (Yeh et al., 2013b).

A connectrometry database was created in DSI Studio with 52 subjects (104 total scans – 52 benchmark and 52 post-training scans). The diffusion data were reconstructed in the MNI space using q-space diffeomorphic reconstruction (Yeh and Tseng, 2011) to obtain the spin distribution function (Yeh et al., 2010). A diffusion sampling length ratio of 1.25 was used. The output resolution in diffeomorphic reconstruction was 2 mm isotropic. The restricted diffusion was quantified using restricted diffusion imaging (Yeh et al., 2017). QA (Yeh et al., 2013b) was extracted as the local connectome fingerprint (Yeh et al., 2016) to be used in the connectometry analysis. The difference between longitudinal scans were calculated by post-training minus pre-training scans. Only increased longitudinal changes were used in the analysis. Diffusion MRI connectometry (Yeh et al., 2016) was used to derive the correlational tractography (a deterministic tractography method) that has longitudinal change of QA correlated with the desired variable.

### 2.4 Statistical analysis

As baseline motor skills and age may represent additional factors affecting WM neuroplasticity [these variables impact motor skill learning in general (Guadagnoli and Lee, 2004; Lillard and Erisir, 2011)], an analysis to determine the relationship between motor performance and neuroimaging results included a two-tailed independent samples t-test between female and male groups to test for significant pre-existing slackline motor skill differences, age differences, and for final slackline skill. The final slackline skill was assessed by the professional trainer from 1 (very bad) to 10 (excellent). Additionally, Spearman’s correlational analysis of mean QA change in the three major highlighted tracts (outlined below) with pre-existing slackline practice (ranked on a 5-point scale: 1 = never attempted, 2 = < 15 min, 3 = 15–30 min, 4 = 30–45 min, 5 = 45–60 min) and Pearson’s correlational analysis of mean QA change in the three major highlighted tracts (outlined below) with age were completed (Supplementary Data 3).

Our correlational tractography analysis used different *t*-statistic thresholds (2.5, 3.0, and 3.5). The *t*-statistic is determined and evaluated to pass the chosen threshold from a non-parametric Spearman partial correlation analysis. This analysis is done at each local connectome to investigate weaker and stronger correlations. Using a range of *t*-thresholds led to higher sensitivity (high true positives at low thresholds) and to higher specificity (high true negatives at high thresholds). Additionally, based on the variety of length thresholds used in previous research (Wang et al., 2019; Hula et al., 2020; Delaparte et al., 2017; Dresang et al., 2021), different length thresholds [15, 20, 25 voxel distances (30, 40, 50 mm)] were used at each *t*-threshold to remove fragmented findings (similar to cluster size in cluster-based analyses). The full in-depth explanation of connectometry as a deterministic tractography method is described in Yeh et al. (2016) (additional explanation of threshold parameters is included in Supplementary Data 5). The cerebellum region was removed from analysis as lower slices in the cerebellum are an area of high level noise in DWI, that is suggested to be removed in DSI Studio tutorials (Li et al., 2022). Additionally, sex focused analyses in the balance group were rerun with the highest thresholds including the cerebellum, showing similar final results (Supplementary Data 4). As the cerebellum is a major area responsible for postural adjustments, future work can focus on the complex WM fiber structure of the cerebellum. Tracts were filtered and selected by topology-informed pruning with 16 iterations (Yeh et al., 2019a). A randomly permuted null distribution is also created with this *t*-threshold. A total of 4000 randomized permutations were applied to the group label to obtain the null distributions of the track length. A false discovery rate (FDR) less than 0.05 indicated a highly confirmative finding. Therefore, only tracts with an FDR < 0.05 were kept. Three different correlational tractography analyses were run. To initially determine differences between the control and intervention group, the correlation between increase in QA and group was determined using a nonparametric Spearman partial correlation, while the effects of age and sex were removed using a multiple regression model. Subsequently, to analyze the effect of biological sex on WM neuroplasticity, an additional interaction variable of Group × Sex was determined for each individual. The correlation between increase in QA and this Group × Sex interaction was then determined, while the effects of age were removed using a multiple regression model. Lastly, to further analyze the effect of biological sex, only individuals in the intervention group (n = 24) were used to determine correlation between increase in QA and sex, while the effects of age were removed using a multiple regression model. Results of the sex effect analysis on the balance group for the highest thresholds [*t*-threshold = 3.5 & length threshold = 25 voxels (50mm)] were clustered by their respective tracts by DSI Studio and only tracts making up more than 10% of the total association of sex and QA increase were highlighted.

## 3 Results

### 3.1 Behavioral data

**FIGURE 1:**
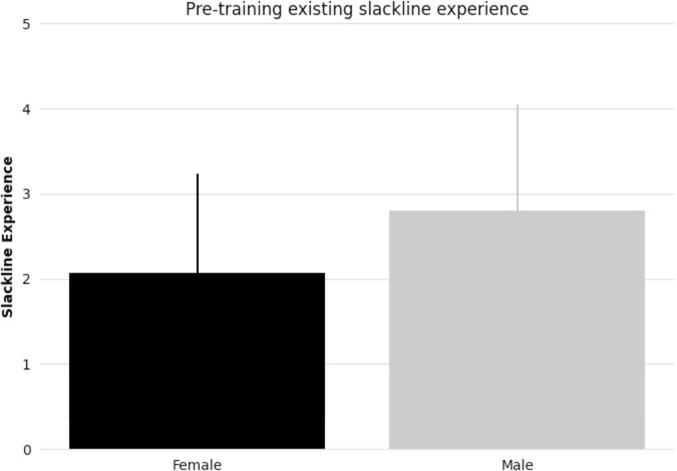
Bar graph comparing female and male mean pre-existing slackline experience (5-point scale: 1 = never attempted, 2 = < 15 min, 3 = 15–30 min, 4 = 30–45 min, 5 = 45–60 min) and standard deviation.

**FIGURE 2:**
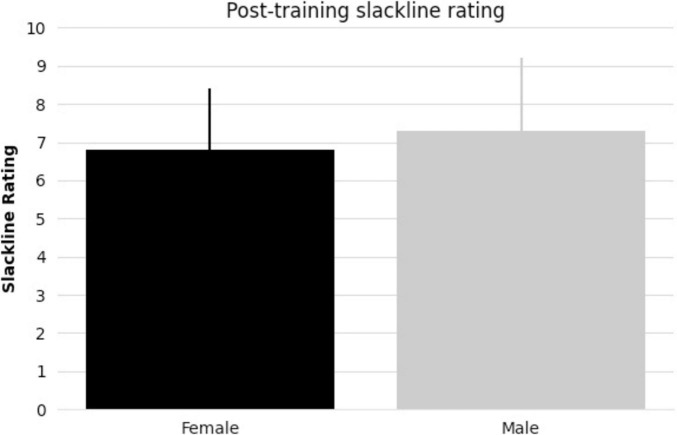
Bar graph comparing female and male mean post-training slackline rating [1 (very bad) to 10 (excellent)] and standard deviation.

All subjects taking part in balance training reached the specified skill level. There were no significant differences between female and male pre-existing slackline skills (*t-statistic* = 1.405, *p* = 0.17, Figure 1). There were also no significant differences between female and male slackline rating after training completion (*t-statistic* = 0.684, *p* = 0.50, Figure 2). Additionally, there were no significant differences between female and male age in the intervention group (*t-statistic* = 0.260, *p* = 0.80).

### 3.2 Neuroimaging

**FIGURE 3:**
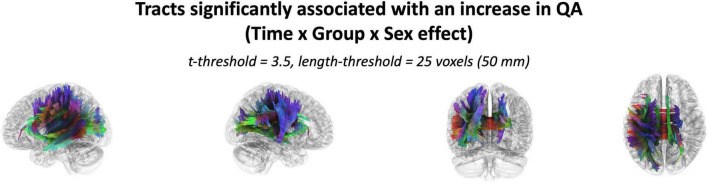
Tracts with increased QA significantly associated with the interaction of Sex and Intervention at a *t*-threshold of 3.5 and length threshold of 25 voxels (50mm). Tractography is rendered by directional colors (red: left-right; green: anterior-posterior; blue: superior-inferior).

The effect of taking part in balance training was significantly associated with an increase in QA at lower thresholds [(*t* = 2.5; Length = 30mm, 40mm, 50mm; FDR < 0.05) & (*t* = 3.0; Length = 30mm; FDR < 0.05)], but this effect was not significantly associated at higher thresholds (Supplementary Data 1). The Interaction effect of Sex × Group was highly associated with increased QA from pre- to post-training across all thresholds (Supplementary Data 2). Results were found in all three fiber types (association, projection, and commissure fibers–Figure 3).

### 3.3 Intervention sex effect

**FIGURE 4:**
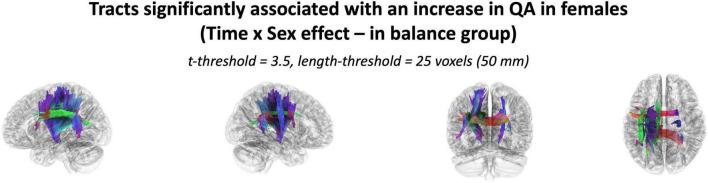
The cluster of tracts with increased QA significantly associated with females in the balance group (Analysis: main effect = biological sex, covariate = age, cohort = balance group). Note only tracts significantly associated with QA increase is shown for females as there were no tracts significantly associated with QA increase in males. Results shown for *t*-threshold of 3.5 and length threshold of 25 voxels (50 mm). Tractography is rendered by directional colors (red: left-right green: anterior-posterior blue: superior-inferior).

**FIGURE 5:**
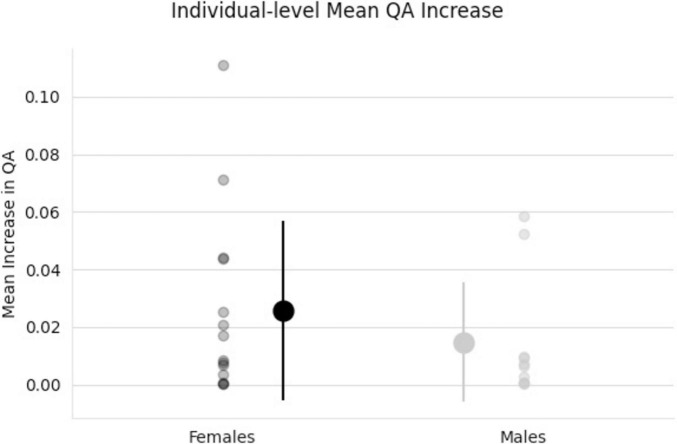
Individual scatter plot data of QA increases. Tracts used for this mean QA comparison are all tracts that showed a significant association with the increase in QA for females from Figure 4. Group means and standard deviations included (middle, larger points) showing larger QA increases for females (black) than males (gray) tracts.

**FIGURE 6:**
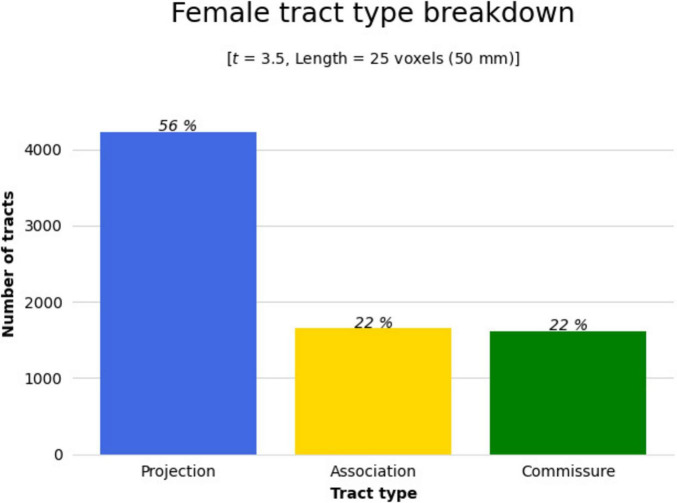
The significant cluster of tracts in Figure 4 (tracts showing a significant association with QA increase in females in the balance group) were clustered by their respective tract types (projection, association, or commissure) by DSI Studio to better determine areas of change. Bar graph breaking down the type of tracts that were shown in Figure 4. Results were majorly found in projection fibers, with significant tracts also noted in association and commissure fibers.

**FIGURE 7:**
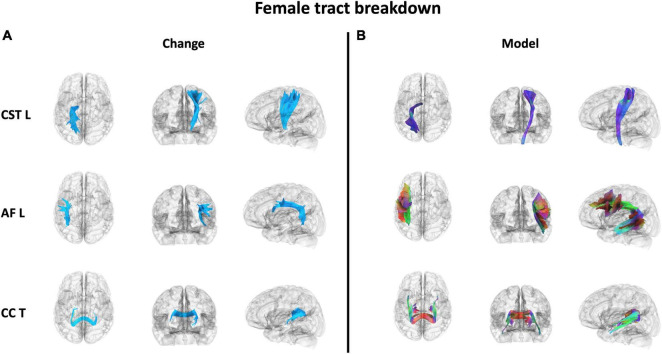
The 3 WM tracts with the highest percentage of tracts associated with increased QA in females with balance training [*t* = 3.5, Length = 25 voxels (50 mm)]. The portion of the tracts associated with the QA increase associated with females **(A)** and the full model tract **(B)**. The model tracts are derived from Yeh et al. (2018) to better portray the full WM tract of interest. CST L = left cortico-spinal tract, AF L = left arcuate fasiculus, CC T = tapetum of corpus callosum.

The effect of participating in balance training was highly associated with an increase in QA for females only, as there were no tracts significantly associated with the increases in QA for males across any thresholds (Table 2). Tracts significantly associated with increased QA in females were similar to the Sex × Group interaction results above (Figure 3 visually compared to Figure 4). Mean QA increase along all the fibers in the cluster of significant tracts from Figure 4 on the individual level is visualized in Figure 5. Tracts having a significant association with an increase in QA in females for the highest thresholds (*t*-threshold = 3.5 & length threshold = 25 voxels; Figure 4) were used for the following tract breakdowns and results (Figures 6, 7 and Table 2).

**TABLE 2:** Number of tracts with a Sex-effect on QA increase (FDR < 0.05).

t-threshold	FEMALE: Tracts @ Length Threshold 15 voxels (30 mm)	MALE: Tracts @ Length Threshold 15 voxels	FEMALE: Tracts @ Length Threshold 20 voxels	MALE: Tracts @ Length Threshold 20 voxels	FEMALE: Tracts @ Length Threshold 25 voxels	MALE: Tracts @ Length Threshold 25 voxels
2.5	18135	0	22919	0	36821	0
3.0	20295	0	111749	0	57795	0
3.5	57111	0	23179	0	7488	0

Results were clustered by their respective tracts, and majorly found in projection fibers, with significant tracts also noted in association and commissure fibers (Figure 6). Only tracts making up more than 10% of the total tracts significantly associated with QA increase for females at the highest thresholds [*t*-threshold = 3.5 & length threshold = 25 voxels (50mm)] were highlighted [the left cortico-spinal tract (CST), arcuate fasciculus (AF), and tapetum of the corpus callosum (CC)] (Table 3 and Figure 7).

**TABLE 3:** Sex-effect specific tract breakdown for females [t = 3.5, Length = 25 voxels (50 mm)].

Tract type	Specific tract	% of tracts
Projection	Corticospinal Tract L	28%
Association	Arcuate Fasciculus L	18%
Commissure	Corpus Callosum Tapetum	12%

Specific tracts that account for less than 10% of the connectometry result are not reported.

### 3.4 Correlations

Spearman’s correlation analysis showed no significant correlations between mean QA increase in the three highlighted WM tracts and pre-existing slackline skill (Supplementary Data 3). Pearson’s correlation analysis showed no significant correlations between mean QA increase in the three highlighted WM tracts and age (Supplementary Data 3).

## 4 Discussion

### 4.1 Summary

The current study characterized widespread QA increases in female human brains associated with balance skill acquisition. This is the first study to our knowledge that employed correlational tractography to investigate WM anisotropy increases associated with motor skill acquisition. The results of our analyses in this younger cohort (mean age = 23.88 years) support our past findings of structural differences between female and male neuroplasticity reported in our previous study with an older cohort (Kirby et al., 2024), similarly showing females exhibiting greater anisotropy increases than males.

### 4.2 Behavioral changes

All subjects that took part in balance training reached the specified skill level in 2 or 3 90-minute sessions. Notably, in line with our previous research findings (Kirby et al., 2024), there were no discernible differences in performance measures between females and males (pre-existing slackline experience, number of sessions to reach skill level, or post-training slackline skill rating). While performance assessments reveal comparable outcomes between females and males in the current study, neuroimaging provides insights into the differences that may underlie these observed behaviors.

### 4.3 Neuroimaging

There were no discernible differences in performance measures between females and males even though QA increases showed differences. This structural evidence aligns with functional studies highlighting variations in fMRI activation patterns between females and males during similar task performance (Hugdahl et al., 2006; Lissek et al., 2007; Gorbet and Sergio, 2007; Andrushko et al., 2023). For instance, Gorbet and Sergio (2007) found no performance discrepancies between females and males in a visuo-motor task of increasing complexity, yet identified differences in fMRI activity. Lissek et al. (2007) showed higher activation in females during simple and complex finger tapping tasks, while both groups achieved comparable error rates. These current findings further suggest that females and males may rely on distinct brain regions or tissue types to achieve comparable outcomes.

Correlational tractography identified several tracts that were identified in the Group × Sex interaction across all connectometry threshold values. However, group effects were only noted at lower thresholds. The importance of biological sex in these results was highlighted by the increase in QA for females across all threshold values, while males failed to show any change. The current study supports the importance of including data that are disaggregated by biological sex in analyses and builds on prior work in this research area (Tannenbaum et al., 2019; White et al., 2021; Kirby et al., 2024; Andrushko et al., 2023; Rechlin et al., 2022; Arnegard et al., 2020). Differences in biological sex in neuroimaging analyses have been shown across diverse techniques (Toschi et al., 2020; Schmithorst et al., 2008; Liu et al., 2010; Björnholm et al., 2017; Gorbet and Sergio, 2007). On a large scale, males demonstrate greater variability in cortical surface area measures, subcortical volume, and cortical thickness, across the brain (Wierenga et al., 2020). Additionally, males have a larger cortical surface area, while females show a greater cortical thickness for many regions (Wierenga et al., 2020; Ritchie et al., 2018). Functionally, females and males show different patterns in GM activation during voluntary motor tasks. Similar to the structural results, functional MRI shows that males demonstrate a larger surface area and higher variability of BOLD signal (Andrushko et al., 2023).

While cross-sectional studies are important, identifying different patterns of change can aid in further understanding neural development, degeneration, and plasticity. Previously, we had suggested possible female versus male differences in WM neuroplasticity during motor learning of a unilateral task (Kirby et al., 2024). The current study builds on this past work in two ways. First, we considered change in young, healthy individuals as they completed balance training. Second, we investigated change across a broad network of WM including projection, association, and commissural tracts. It is important to show these patterns across the lifespan in both younger and older cohorts because of the effect age has on WM (Peters, 2002), as well as the previous MRI work that has been done to identify female and male differences in development and degeneration. Toschi et al. (2020) used advanced multi-shell diffusion MRI to suggest that age-related brain changes related to degeneration begin up to 14 years earlier in males than females. They also suggested that the promyelinating effects of female hormones may protect from age-related vulnerabilities in the myelination process (Bartzokis, 2004). Other work demonstrated sex-specific WM development, with females showing a faster rate to matured WM levels (Wang et al., 2012; Schmithorst et al., 2008). While studies relating WM structural and functional measures to motor performance and skills in females and males are scarce (Schmithorst, 2009; Schmithorst and Holland, 2006, 2007; Schmithorst et al., 2008) determined differing structural and functional neural correlates of cognition between females and males during development. The culmination of studies suggested a greater dependance on WM by females during development, potentially moderated by specific information processing demands. On a more macroscopic scale, it has been reported that females show a greater association of intelligence to WM volume, while GM volume correlates more strongly with intelligence in men on both a global (Gur et al., 1999) and regional scale (Haier et al., 2005) in the brain. Outside of DWI and functional MRI, magnetic resonance spectroscopy studies have also demonstrated WM regions in which N-acetyl-aspartate concentrations correlate with intelligence in females but not in males (Jung et al., 2005; Pfleiderer et al., 2004).

It was important to investigate if the differences in WM neuroplasticity during motor learning occurred outside of the CST, as neuronal activity that is needed to perform a task usually depends on the task demands and increases with parametric changes in task difficulty (Prvulovic et al., 2005; Peters et al., 2020; Wnuk et al., 2018; Andrushko et al., 2021; Danielson et al., 2024; Van Ruitenbeek et al., 2023). Therefore, a full body balance task may recruit more widespread neural activity than the unilateral task from Kirby et al. (2024). Different tasks may lead to more or less observed differences between females and males. Tracts only associated with increases in females were found in all three fiber types. Similar to our previous work (Kirby et al., 2024), females showed an increase in QA associated with motor skill acquisition in projection fibers, mainly the CST (Figures 5, 6). Similarly, select neuroplasticity research during motor learning has focused on unilateral training primarily associated with projection fibers resulting in contralateral brain changes (Lakhani et al., 2016; Kirby et al., 2022; Frizzell et al., 2020, 2021; Sale et al., 2017; Reid et al., 2017). For example, Reid et al. trained participants in a motor task with their left hands and reported increases in FA along the right contralateral CST (Reid et al., 2017). In addition to projection tracts, association tracts showed significant QA increases in females. While most commonly associated with language, the AF (specifically the portion of the AF associated with QA increase in females in the current study – Figure 6) does include connections to the ventral premotor cortex (vPMC). This is a region within the broader premotor cortex, which plays a key role in motor planning and execution (Binkofski and Buccino, 2006; Purves et al., 2001; Kakei et al., 2001). In addition to projection and association tracts, our previous work highlighted the CC (a major commissural tract) as a potential area of significance in motor skill acquisition (Frizzell et al., 2021). This has built on past work associating the CC with motor and visuomotor task activation (Weber et al., 2005; Tettamanti et al., 2002; Fabri et al., 2014; Gawryluk et al., 2011; Mazerolle et al., 2008; D’Arcy et al., 2006; Gawryluk et al., 2014b; Mazerolle et al., 2010; Gawryluk et al., 2014a), as well as, FA measures in this region positively correlating with performance during a motor learning session (Vien et al., 2016).

### 4.4 Clinical relevance

While adding to the female/male neuroscience literature is generally important on a research-level, this is also very important for understanding disease and optimizing rehabilitation. A more focal and consistent neural processing by females would not need an increased speed of signal transmission from a large cortical surface area, but instead would be focused on neural transmission across WM structures for efficient signal transmission (Andrushko et al., 2023; Kirby et al., 2024). As identified in this research, females show a greater increase in anisotropy values in WM during motor learning. This has direct connection to similar results in an older cohort taking part in a different task (Kirby et al., 2024), as well as a more indirect connection (motor research compared to cognition research) to research showing a higher WM dependence by females during development (Schmithorst and Holland, 2006, 2007; Schmithorst, 2009). Speculatively, the current study adds to this by potentially suggesting a higher WM dependence in motor skill acquisition as well. Although this may be beneficial in healthy, neurologically intact populations, to facilitate efficient nervous signal transmission, this approach may be detrimental in the presence of a neurological injury. For example, with stroke and MS, injury to WM interrupts axon signal transmission, resulting in impairment. As the incidence and presentation of many neurological conditions and movement disorders differ between sexes (Meoni et al., 2020), a higher WM dependance may be the reason behind more severe result of WM injury and disease in females (Tomita et al., 2015; Kim et al., 2010; Phan et al., 2019; Persky et al., 2010; Golden and Voskuhl, 2017; Voskuhl, 2020; Koch-Henriksen and Sørensen, 2010).

Along with WM disease, traumatic brain injury (TBI) commonly causes WM damage (Guerrero-Gonzalez et al., 2022; Wright et al., 2021; Narayana, 2017). Studies have highlighted varying recovery trajectories and outcomes for females vs. males following TBI (Gupte et al., 2019; Mikolić et al., 2021; Haynes and Goodwin, 2023). Furthermore, hormonal influences, particularly progesterone which is commonly associated with females, may be neuroprotective when used as a TBI treatment (Wright et al., 2007). As hormonal fluctuations have been shown to effect neuroplasticity (Catenaccio et al., 2016; Pawluski et al., 2016; Barha and Galea, 2010; Saleki et al., 2022), common sex-related hormones like testosterone, estrogen, and progesterone may be the basis for observed female and male differences. Understanding WM changes is important for exploring targeted therapeutic strategies that harness neuroplasticity for improved recovery. This also extends to sex differences in neonatal brain injury and repair, as infant sex can play a role in disease onset, course and resolution (Rosenkrantz et al., 2019). Although many explanatory factors have been identified in sex-specific WM injury outcomes, incorporating learning-induced WM changes into this phenomenon should be discussed.

### 4.5 Future work and limitations

The current study focused on identifying greater anisotropy increases in females over males in a motor skill acquisition paradigm. This was mainly because previous connectometry analyses focused on QA decreases associated with degeneration (Moshchin et al., 2023; Huang et al., 2023) and increases in QA associated with cognitive rehabilitation (Brownsett et al., 2024). However, prior work identified decreases in anisotropy as helpful for neuroplasticity. For example, our past work identified a decrease in anisotropy in older males during motor skill acquisition (Kirby et al., 2024). Additionally, Taubert et al. (2010) investigated neuroplasticity during balance training, observing reductions in FA associated with learning. FA and other anisotropy measures have only demonstrated low sensitivity to a single neurobiological process (Jones et al., 2013) making the ability to characterize the exact mechanism behind neuroplasticity changes a limitation of the current study. Future work can include both traditional diffusion tensor imaging-based metrics along with complex metrics such as QA (tractography comparison in Yeh et al. (2013b)) to aid in better understanding neurophysiology associated with motor skill acquisition changes. As Fields (2015) has pointed out, conduction velocity along WM is changed to optimize timing of information transmission through neural circuits. Therefore, attenuated increases in myelination or anisotropy values of a certain WM path may occur to increase efficiency of a network. Future work should better elucidate what increase/decrease in anisotropy is specifically showing in regard to neuroplasticity as decreases may also be positive in some instances.

The current study highlights the importance of different brain tissue types in females and males. As training-induced alterations in different types of brain tissue likely operate on different time-scales and may further change in follow-up assessments (Weber et al., 2019; Zatorre et al., 2012), the focused hypothesis on the effect of motor skill acquisition at two timepoints is a limitation of the current study. It is important to better understand the timing of these changes, especially in both GM and WM regions. Speculatively, females and males may take part in similar neurophysiological changes, but at different timescales. These suggestions warrant further investigation into the biological implications underlying these observations.

As this study used publicly available data, our analysis was restricted to the previously collected data. The current study dataset was unique in terminating training and initiating the post-training timepoint once an individual passed a specified behavioural performance test, indicating they had reached a measurable skill level. However, the nature of the task makes it difficult to quantitatively measure pre-existing skills or comparison of final skill level outside of the passing test, reflected in the discrete behavioral measures used. Greater challenges in a training paradigm may lead to greater changes in WM measures (Lakhani et al., 2016). Therefore, it is important for future work to measure a wide variety of pre-existing motor skill competencies and to note that complexity, task-specific goals, and baseline motor skill competency represent additional factors that may affect WM neuroplasticity, as these have previously been shown to impact motor skill learning in general (Guadagnoli and Lee, 2004; Wulf et al., 2010).

Finally, as mentioned above, the most studied sex-based hormones, estrogen and testosterone, are known to affect neuroplasticity (Catenaccio et al., 2016; Pawluski et al., 2016; Barha and Galea, 2010; Saleki et al., 2022). Therefore, it is important for future work not only to include the effect of biological sex, but also to define how hormones effect WM changes. There are other data suggesting that hormonal balance can affect neuroplasticity even at the cellular level (Catenaccio et al., 2016; Laube et al., 2020). However, given the current analysis, consideration of these effects is beyond our scope. Future work could consider how these factors interact.

## 5 Conclusion

The current study suggests that female and male differences in WM neuroplasticity during motor skill acquisition may be detectable using MRI-derived DWI measures and connectometry. The current study builds on past work (Kirby et al., 2024) by considering change in young, healthy individuals as they completed balance training and by investigating change across a broad network of WM including projection, association, and commissural tracts. Importantly, we discovered differences in WM changes for females versus males despite both groups reaching a similar level of balance motor skill. These findings suggest that there are distinct patterns of change that take place in female versus male brains to support motor skill acquisition. We provide further support for the importance of including sex-based analyses, as females and males may exhibit differences in neural mechanisms (Toschi et al., 2020; Kopec et al., 2018; Rogojin et al., 2023). As pathophysiology of many illnesses and their resulting rehabilitation differ between females and males, our data suggest that future work should consider how differences in biological sex affect patterns of both structural and functional change associated with motor learning. These differences in tissue dependance likely have important clinical implications for understanding impairment severity and rehabilitation strategies for each sex independently.

## Data Availability

Publicly available datasets were analyzed in this study. This data can be found here: https://doi.org/10.18112/openneuro.ds003138.v1.0.1.
