# Supplementary material for: Sex differences in patterns of white matter neuroplasticity after balance training in young adults
Source: Front Hum Neurosci. 2024 Aug 27;18:1432830. doi: 10.3389/fnhum.2024.1432830 (PMC11383771; doi:10.3389/fnhum.2024.1432830)
Supplement: Supplementary file 1 [file Data_Sheet_1.docx]

Supplementary Material

# Supplementary Data 1 – *Intervention Effect*

**
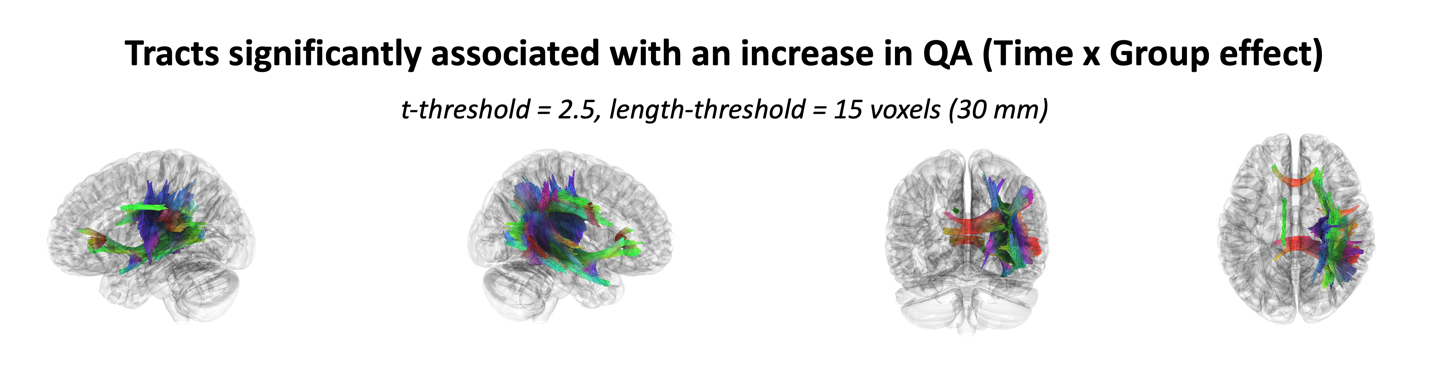
**

**Supplemental Figure 1:** Tracts with an increased QA from pre to post significantly associated with taking part in balance training at a *t*-threshold of 2.5 and length threshold of 15 voxels (30mm). Tractography is rendered by directional colours (red: left-right green: anterior-posterior blue: superior-inferior).

**Supplemental Table 1:** Number of tracts with an intervention group effect on QA increase (FDR < 0.05):

| ***t*-threshold** | ***Length Threshold 15 voxels*** | ***Length Threshold 20 voxels*** | ***Length Threshold 25 voxels*** |
| --- | --- | --- | --- |
| ***2.5*** | 14876 | 2531 | 183 |
| ***3.0*** | 857 | - | - |
| ***3.5*** | - | - | - |

# Supplementary Data 2 *– Group × Sex interaction*

**Supplemental Table 2:** Number of tracts with a Group × Sex interaction on QA increase (FDR < 0.05)

| ***t*-threshold** | ***Length Threshold 15 voxels*** | ***Length Threshold 20 voxels*** | ***Length Threshold 25 voxels*** |
| --- | --- | --- | --- |
| ***2.5*** | 42586 | 16480 | 20393 |
| ***3.0*** | 25402 | 25339 | 112302 |
| ***3.5*** | 73193 | 197718 | 103958 |

# Supplementary Data 3 *– Correlations*

Spearman’s correlation analysis showed no significant correlations between mean QA increase in the three highlighted WM tracts and pre-existing slackline skill (Supplemental Table 3).

**Supplemental Table 3:** Pre-existing slackline skill and mean tract increase in QA. CST_L = left cortico-spinal tract, CC_T = corpus callosum tapetum, AF_L = left arcuate fasciculus

| **White Matter Tract** | **Spearman’s Correlation Coefficient (significance)** |
| --- | --- |
| *CST_L* | -0.25 (*p* = 0.23) |
| *CC_T* | -0.22 (*p* = 0.30) |
| *AF_L* | -0.18 (*p* = 0.39) |

Pearson’s correlation analysis showed no significant correlations between mean QA increase in the three highlighted WM tracts and age (Supplemental Table 4).

**Supplemental Table 4:** Age and mean tract increase in QA. CST_L = left cortico-spinal tract, CC_T = corpus callosum tapetum, AF_L = left arcuate fasciculus

| **White Matter Tract** | **Pearson’s Correlation Coefficient (significance)** |
| --- | --- |
| *CST_L* | 0.21 (*p* = 0.33) |
| *CC_T* | 0.31 (*p* = 0.15) |
| *AF_L* | 0.20 (*p* = 0.34) |

# Supplementary Data 4 *– Effect of sex in balance group (cerebellum included)*

Visually, it can be noted that both Supplemental Figure 2a and 2b are very similar, showing the inclusion or exclusion of the cerebellum in the analysis, did not affect the results. This pattern stayed true for all analyses with the cerebellum included or excluded. We do acknowledge that the cerebellum is a major area responsible for postural adjustments. Therefore, future work can focus on the complex WM fiber structure of the cerebellum.


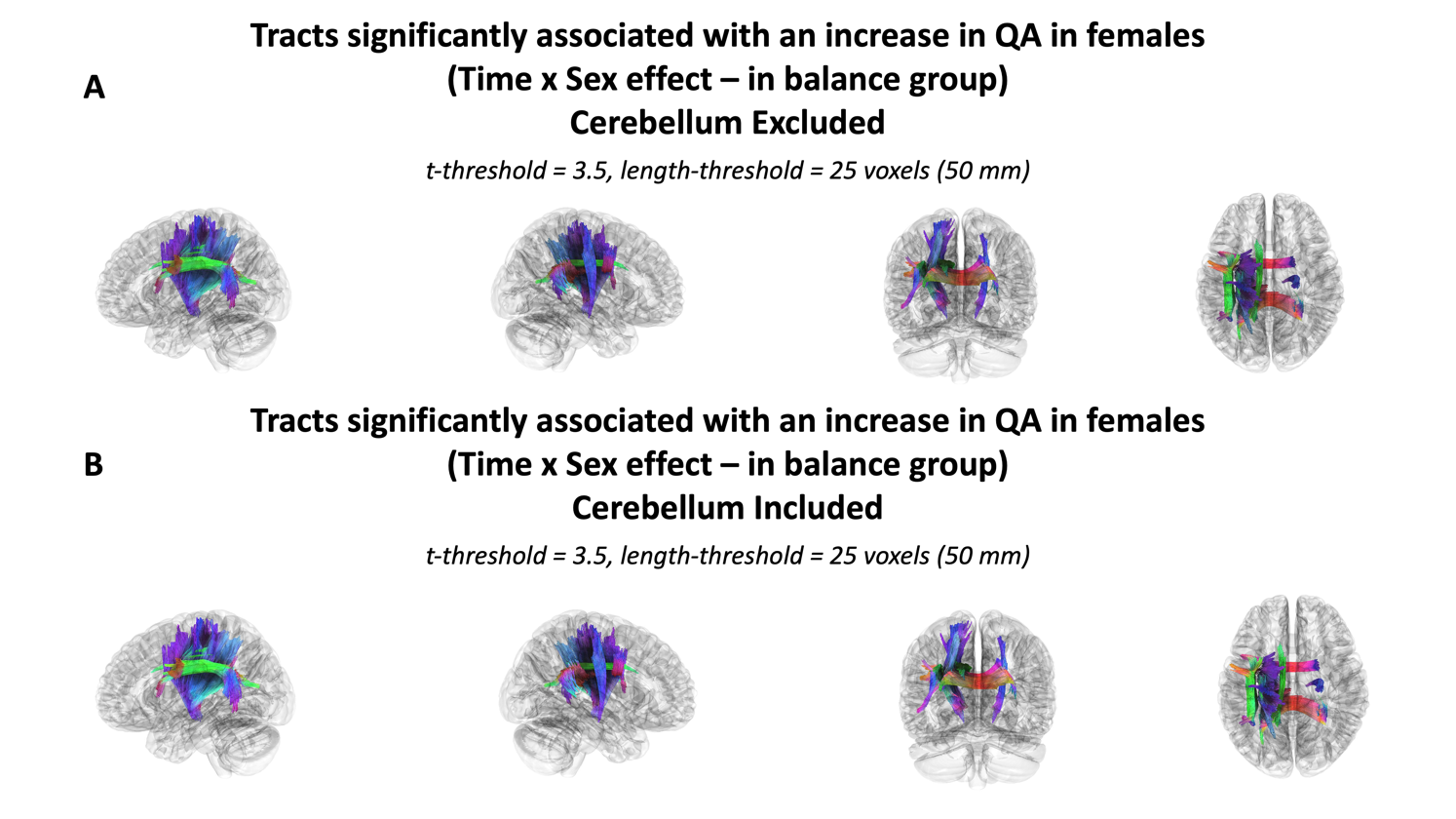


**Supplemental Figure 2:** Tracts with increased QA significantly associated with the effect of Sex at a *t*-threshold of 3.5 and length threshold of 25 voxels (50mm) in the balance group [**A**: with cerebellum excluded in analysis – same as Figure 4; **B**: with cerebellum included in analysis]. Note only tracts significantly associated with QA increase is shown for females as there were no tracts significantly associated with QA increase in males. Tractography is rendered by directional colours (red: left-right green: anterior-posterior blue: superior-inferior).

# Supplementary Data 5 *– Length and t-threshold explanation*

Correlational tractography analysis is based in connectometry (Yeh, Badre, and Verstynen 2016). The *t*-statistic of the correlation between anisotropy and variable of interest is computed across each voxel of the brain. Then, deterministic fiber tracking (Yeh et al. 2013) is applied using a *t*-threshold (a range of *t*-thresholds leads to higher sensitivity - high true positives at low thresholds - and higher specificity - high true negatives at high thresholds) to map correlational tractography. As a result, correlational tractography shows the location where pathways have diffusion metrics substantially correlated with the study variable. Additionally, similar to the cluster analysis of fMRI, where a permutation test can be applied to examine the size of the clusters, the random permutation test can be applied to examine the size of clusters. The random permutation will generate a length distribution of correlational tractography, corresponding to the null hypothesis, while the length distribution of non-permuted conditions will be derived by applying bootstrap resampling without permutation. The two distributions (the permuted distribution and the non-permuted distribution) will be compared by computing their area under the curve, given a length threshold. Different length thresholds (15, 20, 25 voxel distances) were used at each *t*-threshold to show the effect of more strict tracking parameters, as well as to remove fragmented findings. Further explanation of correlation tractography and the techniques underlying the analysis method can be found at https://dsi-studio.labsolver.org and references: (Yeh et al. 2013; Yeh, Badre, and Verstynen 2016; Yeh, Tang, and Tseng 2013; Yeh, Wedeen, and Tseng 2010; Yeh, Panesar, et al. 2019; Yeh, Zaydan, et al. 2019; Yeh et al. 2017; 2021).
